# *A*CrF_3_ Jahn–Teller-Distorted Fluoroperovskites: Expanding to RbCrF_3_ and CsCrF_3_

**DOI:** 10.1021/acs.inorgchem.5c02668

**Published:** 2025-09-04

**Authors:** Øystein S. Fjellvåg, Heesoo Park, Fabien Veillon, Jike Lyu, Marisa Medarde, Salah B. Amedi, Fabian L. M. Bernal, Helmer Fjellvåg, Bjørn C. Hauback, Bruno Gonano

**Affiliations:** † Department for Hydrogen Technology, 11312 Institute for Energy Technology, PO Box 40, Kjeller NO-2027, Norway; ‡ Chemistry Department and Center for Material Science and Nanotechnology, 6305 University of Oslo, Oslo NO-0315, Norway; § 69240 Laboratory Crismat, UMR6508 CNRS, Normandie University, ENSICAEN, UNICAEN, 6 bd Maréchal Juin,cedex 4, Caen 14050, France; ∥ 28498 PSI center for Neutron and Muon Sciences, Villigen-PSI CH-5232, Switzerland; ⊥ Division for Research, Dissemination and Education, IT-department, 6305 University of Oslo, Oslo N-0316, Norway; # CAS Key Laboratory of Magnetic Materials and Devices, Ningbo Institute of Materials Technology and Engineering, Chinese Academy of Sciences, Ningbo 315201, China

## Abstract

The Jahn–Teller effect significantly affects the CrF_6_ octahedra in Cr­(II) fluoroperovskites. Here, we report the synthesis, crystal structures, and magnetic properties of RbCrF_3_ and CsCrF_3_, thereby completing a comprehensive investigation of the *A*CrF_3_ fluoroperovskites. Powder samples are prepared using a wet-chemical method, which allows stabilization of Cr­(II). RbCrF_3_ predominantly adopts the *P*4/*mbm* space group; however, the absence of the expected (211) peak suggests a limited correlation length, and we demonstrate that this is a result of stacking faults in the rotation of the distorted CrF_6_ octahedra along the *c*-axis. We estimate around 5–10% of *I*4/*mcm*-type stacking in *P*4/*mbm* for RbCrF_3_. In contrast, CsCrF_3_ showcases a two-phase coexistence of *I*4/*mcm* and *P*4/*mbm* polymorphs and, as such, parallels to KCuF_3_. We discuss and highlight the interplay between the impact of *A*-site cations and the Jahn–Teller effect acting on CrF_6_ octahedra. Antiferromagnetic interactions dominate the paramagnetic regime, and RbCrF_3_ and CsCrF_3_ both have a Weiss temperature of −12 K, associated with Néel temperatures of 50 and 47 K, respectively. The effective paramagnetic moments of 4.65 and 4.68 μ_B_ for RbCrF_3_ and CsCrF_3_ are in good agreement with *S = 2* spin configuration.

## Graphical abstract

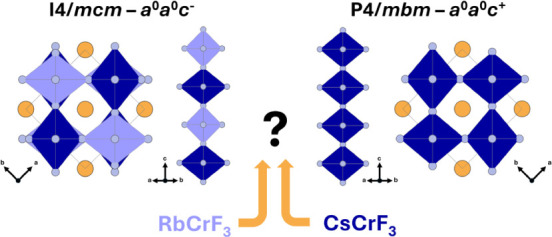

## Introduction

The cooperative Jahn–Teller effect in transition metal ions arises from the interplay between electronic degeneracy and lattice vibrations.^1–3^ When electron–phonon interactions are strong, they lift the orbital degeneracy, stabilize a specific orbital configuration, and distort the metal–ligand octahedra. These deformations occur collectively throughout the crystal, resulting in long-range structural distortions. This selective orbital stabilization and structural distortion result in orbital ordering, a phenomenon closely linked to a variety of important and often highly tunable physical properties. Materials containing Jahn–Teller active ions are particularly intriguing because of the complex interplay, structurally and electronically.^4^

Jahn–Teller active *d* ^4^ cations significantly influence structural properties through distortions and octahedral tilting, driven by both steric factors and cooperative Jahn–Teller effects. This feature is exemplified for Mn^3+^ for the layered materials *A*MnF_4_ (*A* = Na, K, Rb, Cs).^5^ NaMnF_4_ exhibits a highly distorted monoclinic structure (space group *P*2_1_/*a*) with significant octahedral tilting and noncollinear antiferromagnetic ordering.^6^ In contrast, KMnF_4_ and RbMnF_4_ maintain a monoclinic symmetry but show reduced distortions, with KMnF_4_ displaying a magnetic canting angle of around 17^◦^, whereas RbMnF_4_ orders collinearly.^7,8^ The large ionic radius of Cs allows CsMnF_4_ to adopt a tetragonal structure (*P*4/*nmm*) and transition to ferromagnetic order below 19 K.^7,9^ This illustrates how the interplay between Jahn–Teller ions and volume effects, as demonstrated by the various *A*-site substitutions, dramatically affects structural and magnetic properties.

In contrast to Mn^3+^, the isoelectronic Cr^2+^ has been less explored, although the Jahn–Teller effect strongly influences both cations. This limited exploration is primarily due to the challenges in synthesizing Cr­(II) compounds with the instability toward oxidation and the strenuous chemistry of Cr­(II). However, in recent years, successful synthesis methods for *A*CrF_3_ fluoroperovskites have provided a route to stabilize Cr^2+^ materials during crystallization, allowing these to be examined under scrutiny.^10–12^

NaCrF_3_ (Glazer tilt *a* ^–^ *b* ^–^ *c* ^–^) is a triclinic fluoroperovskite (*P*1̅).^10,11^ The crystal structure is highly distorted by the combination of the relatively small Na cation (*r*_ionic_ = 139 pm for 12-coordinated Na^+^) and the Jahn–Teller active Cr­(II) cation. In NH_4_CrF_3_, the larger ${\mathrm{NH}}_{4}^{+}$ -cation (*r*_ionic_ = 167 pm^13^ stabilizes a tetragonal (*P*4_2_/*mbc*) structure at room temperature, but displays buckled Cr–F–Cr bonds due to additional hydrogen bonding.^12^ NH_4_CrF_3_ undergoes an order–disorder transition at 405 K, during which the ${\mathrm{NH}}_{4}^{+}$ groups become disordered, and the Cr–F–Cr bonds straighten. KCrF_3_ (*r*_ionic_ = 164 pm for K^+^) adopts a tetragonal crystal structure (*I*4/*mcm*) at room temperature, but becomes monoclinic below 250 K.^14,15^ The tetragonal structure displays antiferrodistortive $3d_{3x^{2}-r^{2}}$ and $3d_{3y^{2}-r^{2}}$ orbital ordering in the *ab*-plane and is similar to that of the *a*-type polymorph of KCuF_3_.^14,16^

The difference between the *a*- and *d*-type polymorphs is particularly important for this work and is illustrated in Figure 1. In the *P*4/*mbm* and *I*4/*mcm* polymorphs, the chromium coordination polyhedra are elongated octahedra with two long, two medium, and two short Cr–F bonds. The stacking of these corner-sharing octahedra differs between the two polymorphs. For the *P*4/*mbm* polymorphs, it has C-type orbital ordering and a Glazer tilt of *a* ^0^ *a* ^0^ *c* ^+^, which implies that the distorted CrF_6_-octahedra are stacked along the *c*-axis without rotation (Figure 1). For the *I*4/*mcm* polymorph, we have a G-type orbital ordering and a Glazer tilt of *a* ^0^ *a* ^0^ *c* ^–^. In that case, the distorted CrF_6_ octahedra are stacked along the *c*-axis with a 90^◦^ rotation.

**Figure 1:**
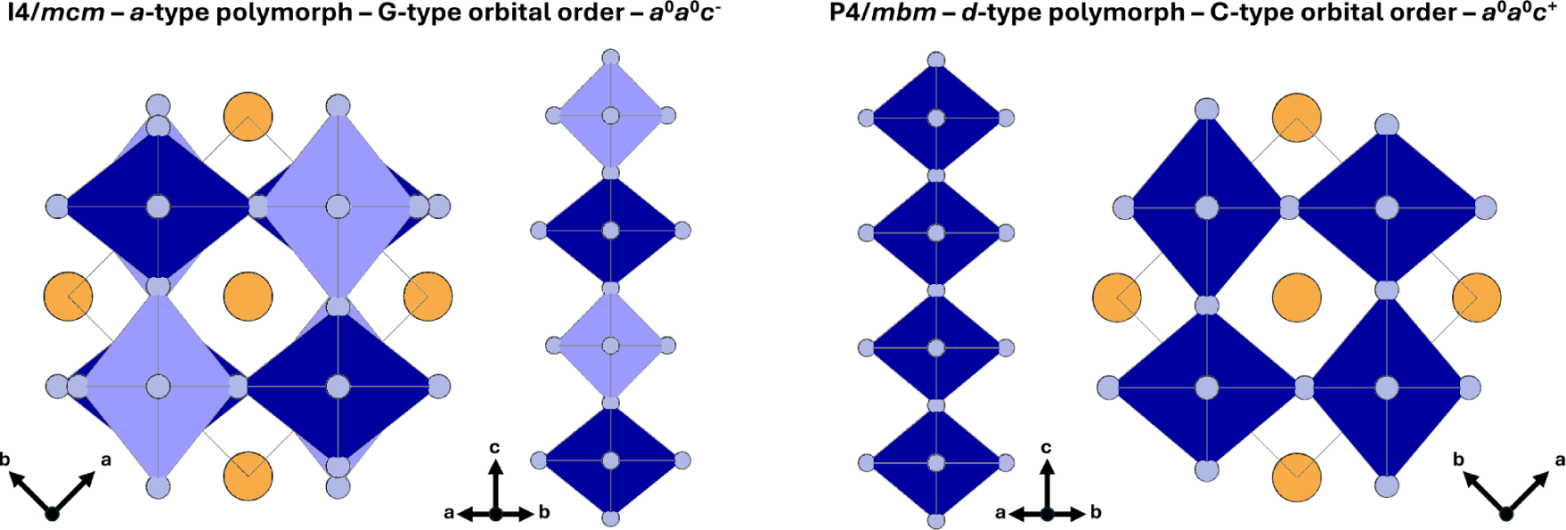
Crystal structure and stacking of Jahn–Teller distorted CrF_6_ octahedra in blue in *I*4/*mcm* and *P*4/*mbm* polymorphs. *A*-site atoms are in orange. In the *I*4/*mcm* polymorph, the CrF_6_ octahedra are rotated 90^◦^ around [001]-direction when stacked along the *c*-axis, while in the *P*4/*mbm* polymorph, the octahedra are not rotated. Octahedra rotated by 90^◦^ are shaded for the *I*4/*mcm* polymorph to highlight the rotation. Due to the difference in stacking, the *I*4/*mcm* polymorph has a *c*-axis that is twice as long as the *P*4/*mbm* polymorph.

This work extends the series of Cr­(II) fluoroperovskites to include two new members, RbCrF_3_ and CsCrF_3_, and explores the impact of variable *A*-site radii. The ionic radii of Rb and Cs are 172 and 188 pm, respectively. The compounds are prepared through a wet-chemical method previously described.^10–12^ Characterization of the materials is done through high-resolution X-ray diffraction and magnetometry, supported by DFT calculations. We finally discuss the structural and physical properties of the entire group of *A*CrF_3_ fluoroperovskites. We consider this as the entire group, although TlCrF_3_ potentially could exist. However, we avoid it due to the toxicity associated with Tl. LiCrF_3_ is expected to adopt a different structure type as no Li*M*F_3_ compound adopts a perovskite-related structure and Li is not expected to show 12-fold coordination.

## Experimental Section

Powder samples of RbCrF_3_ and CsCrF_3_ were synthesized according to the procedure established by Bernal et al.^10–12^ All work was carried out under strictly inert conditions on a Schlenk line and with degassed solvents. The preparation of chromium­(II) acetate is described in detail in refs. 10–12

RbCrF_3_ was synthesized by adding 2 mL H_2_O and 5 mL methanol to 0.5 g of chromium­(II) acetate in a polycarbonate vial and heating to 70 ^◦^C. 1.3 g of RbF (Sigma-Aldrich, 99.8%) dissolved in 2 mL H_2_O was also heated to 70 ^◦^C and transferred to the chromium­(II) acetate solution under vigorous stirring, during which the product precipitated. Similarly, CsCrF_3_ was synthesized from 0.5 g chromium­(II) acetate with 2 mL H_2_O and 5 mL methanol, and 2 g of CsF (Sigma-Aldrich, 99.9%) dissolved in 2 mL H_2_O. The products were decanted, washed three times with methanol, filtered, and dried under vacuum.

The synthesis of NaCrF_3_, KCrF_3_, and NH_4_CrF_3_ fluoroperovskites used in this work are presented in detail in refs. 10–12 .

High-resolution powder X-ray diffraction data were collected at the Swiss-Norwegian beamlines, BM31, at the European Synchrotron Radiation Facility. Powder samples of RbCrF_3_ and CsCrF_3_ were packed in 0.5 mm diameter quartz capillaries, and data were collected using a 2D PILATUS2M detector and a wavelength of 0.25448 Å. The data were reduced using the Bubble software.^17^ Rietveld refinements and stacking fault calculations were carried out in TOPAS.^18,19^ The stacking faults formalism of Topas is based on the DIFFaX formalism, where stacking faults are introduced by modifying the stacking sequence within a large simulation cell.^19,20^ The stacking faults were simulated with probabilities between 0.05% and 100% with the peak shape established from the Rietveld refinements of RbCrF_3_ (note, 0% is not possible to simulate in TOPAS). Ten sequences with 400 stacks per sequence were used for the calculations.

Magnetometry experiments were performed with a Magnetic Property Measurement System XL (MPMS, Quantum Design) on polycrystalline powder samples. Temperature-dependent DC magnetic susceptibility χ­(*T*) measurements were conducted between 2 and 350 K under zero field-cooled/field-cooled (500 Oe) conditions (ZFC and FC, respectively). Curie–Weiss fits were performed between 150 and 300 K. Isothermal field-dependent data *M*(*H*) were collected at 5 K between −50 kOe and 50 kOe.

For optical measurements, powder samples were mounted in sealed 1 mm capillaries and measured using a FLAME-S spectrometer from OceanOptics, equipped with a white diode as the light source. The incident light was directed perpendicularly onto the sample surface, while the detector was positioned at a 45^◦^ angle relative to the incident beam. Before each measurement, the instrument was calibrated using a barium sulfate (BaSO_4_) reference sample. Absorbance spectra were derived from reflectance data using the extended Kubelka–Munk method.^21^

DFT calculations were performed using the Vienna Ab initio Simulation Package (VASP)^22,23^ using the projection-augmented wave approach^24,25^ within the PBE (Perdew–Burke–Ernzerhof) general gradient approximation (GGA).^26,27^ Structural optimizations and total energy assessments for all configurations were executed with 20-atom unit cells and an *A*-type antiferromagnetic structure, employing a Monkhorst–Pack *k*-mesh of (6 × 6 × 6). The cutoff energy for the plane wave was set at 540 eV. Hubbard-corrected calculations have earlier shown results compatible with experiments for chromium­(II).^11,28^ The Coulomb interaction parameter *U* was hence set to 9 eV, and the exchange interaction parameter *J*_H_ to 0.88.

## Results and Discussion

### Structural Chemistry

The as-synthesized powder samples of RbCrF_3_ and CsCrF_3_ were pale blue, similar to the other *A*CrF_3_ (*A* = Na, K, NH_4_), indicating a divalent state of chromium. The electronic state for the Jahn–Teller distorted Cr­(II) was highlighted through optical measurements. The optical absorption data show three broad spin-allowed and two sharp spin-forbidden transitions associated with the splitting of the *t*_2*g*_ and *e*_*g*_ orbitals due to the Jahn–Teller effect^11^ (see the Supporting Information). The energies for spin-forbidden transitions are compared for the *A*CrF_3_ fluoroperovskites in Figure 2. Although the transitions are very similar in energy and in agreement with a Cr­(II) oxidation state, there is a small decrease in energy with increasing unit cell volume. This is consistent with a decreasing crystal field due to the unit cell expansion, as induced by the size of the *A*-site cations.^29,30^

**Figure 2:**
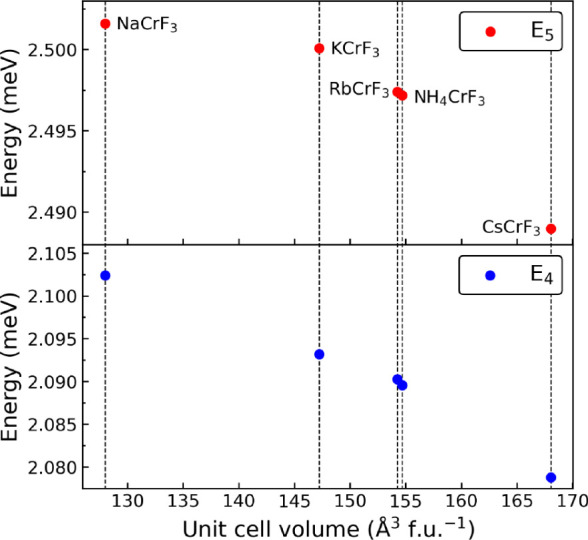
Spin-forbidden E_4_ and E_5_ excitations from optical absorption spectroscopy of *A*CrF_3_ (*A* = Na, K, Rb, Cs, NH_4_) as a function of unit cell volume.

The X-ray diffraction patterns at room temperature of RbCrF_3_ and CsCrF_3_ indicate that both compounds are tetragonal perovskites. Furthermore, a weak, broad peak appears between (200) and (112) (see the Supporting Information) at 2.05 Å^–1^ and 2.0 Å^–1^ for RbCrF_3_ and CsCrF_3_, respectively. This additional peak is more prominent for RbCrF_3_, and is believed to be due to a secondary cubic phase caused by a minor oxidation of the samples. Based on Rietveld refinements, we calculate a mass fraction of 7.8(8) % for a cubic phase (*Pm*3̅*m*) with a tentative composition Rb_1–*x*_CrF_3_. However, the composition could not be refined due to peak overlap and the small amount of the phase. For CsCrF_3_, the amount of the cubic phase was too small to be included in the final refinements.

There exist at least two tetragonal polymorphs for the Jahn–Teller distorted perovskites; *I*4/*mcm* (*a*-type) and *P*4/*mbm* (*d*-type), see Figure 1. The two polymorphs can be distinguished based on the weak Bragg reflections at 2.28 Å^–1^ (*P*4/*mbm*), 2.40 Å^–1^ (*I*4/*mcm*), and 2.75 Å^–1^ (*P*4/*mbm*) for RbCrF_3_ and at 2.19 Å^–1^ (*P*4/*mbm*), 2.32 Å^–1^ (*I*4/*mcm*), and 2.67 Å^–1^ (*P*4/*mbm*) for CsCrF_3_. The main Bragg peaks are not influenced by the different octahedral stacking of the polymorphs.

We compare the experimental data with the calculated diffraction patterns for the two polymorphs in Figure 3. For RbCrF_3_, it is clear that the (210)-peak from *P*4/*mbm* at 2.28 Å^–1^ is present. However, the expected (211) peak at 2.75 Å^–1^ for *P*4/*mbm* is not as intense as the calculated diffraction peak, and is close to absent. We further note that the possible (211)-peak at 2.40 Å^–1^ from *I*4/*mcm* is absent. This comparison suggests that the crystal structure of RbCrF_3_ is fairly well described by space group *P*4/*mbm*.

**Figure 3:**
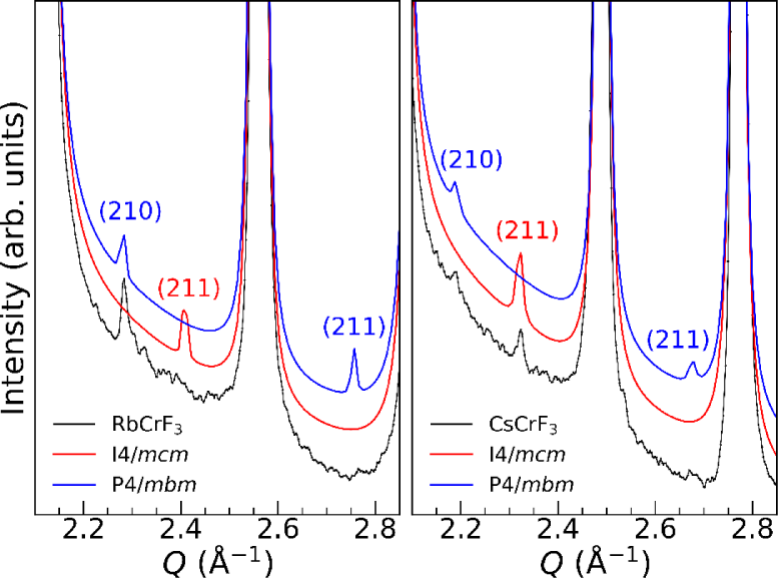
Comparison of experimental and calculated diffraction profiles for RbCrF_3_ and CsCrF_3_ in space groups *I*4/*mcm* (red) and *P*4/*mbm* (blue) with indices for the weak peaks. The calculated profiles are offset for clarity.

The (211)-peak at 2.75 Å^–1^, which is related to the stacking of the CrF_6_ octahedra in RbCrF_3_, is nearly absent. This may indicate a limited correlation length in the bulk material, as well as the presence of stacking faults. To evaluate this, we constructed a stacking fault model and calculated the diffraction profiles for different stacking fault probabilities. The probability represents the likelihood of a 90^◦^ rotation of the distorted CrF_6_ octahedra when stacked along the *c*-axis. This means that 100% correct stacking corresponds to the *I*4/*mcm* polymorph, while 0% correct stacking corresponds to the *P*4/*mbm* polymorph, see Figure 1. As the stacking faults are just a small variation in the position of the F atoms and the main body of the structure is not influenced by the stacking faults (Figure 1), the strong Bragg peaks are unaffected, while the weak Bragg peaks distinguishing the two polymorphs are strongly affected. The calculated diffraction profiles for different probabilities are presented along with experimental data in Figure 4. Already for high probabilities, the (211) peak of the *I*4/*mcm* gradually disappears with decreasing stacking fault probabilities and is weak and broad for a probability of 90–95% and is not visible at or below 80%.

**Figure 4:**
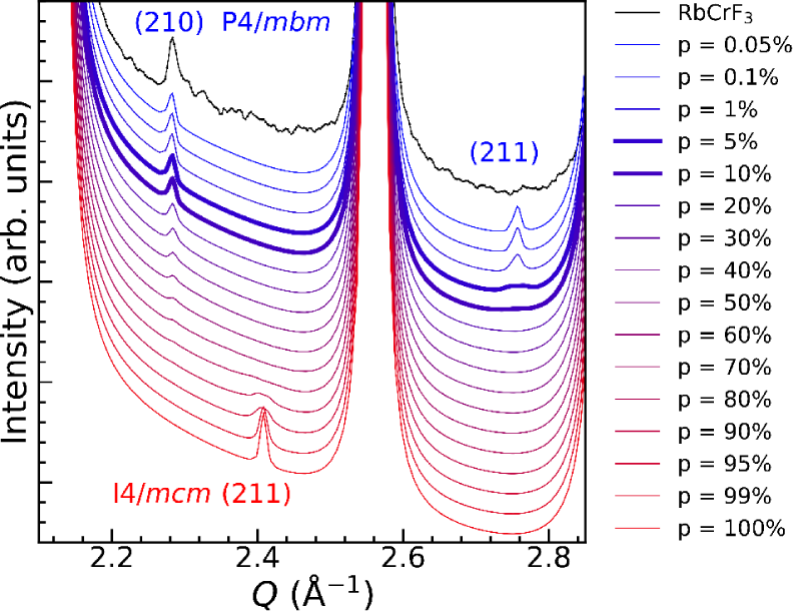
Comparison of experimental and simulated diffraction profiles for RbCrF_3_ with different stacking fault probabilities. 0% corresponds to *P*4/*mbm* and 100% corresponds to *I*4/*mcm*. Five and 10% are shown with thicker lines.

As the stacking fault probability increases from 0%, the (210) and (211) Bragg peaks from the *P*4/*mbm* phase gradually diminish, Figure 4. Notably, the (211) peak decreases in intensity more rapidly than the (210) peak. At a stacking fault probability of 5%, the (211) peak becomes very broad and weak, while the (210) peak remains clearly visible. The (210) peak can still be observed up to 70–80% fault probability, whereas the (211) peak is only discernible at and below 5%. Interestingly, when *p* = 5 and 10%, the simulated diffraction profile aligns well with our experimental diffraction pattern, suggesting that RbCrF_3_ contains a concentration of approximately 5–10% stacking faults. Since the stacking fault concentration is determined from a few weak Bragg peaks, the *R*_*wp*_ values for different stacking fault concentrations are too similar to reliably distinguish between them. As such, Rietveld refinements with and without stacking faults yield quantitatively similar results, and the method cannot be used to determine a clear minimum. Thus, comparison with simulated patterns as shown in Figure 4 is the best way to assess the concentration of stacking faults in RbCrF_3_.

For CsCrF_3_, the peaks at 2.19 Å^–1^ and 2.67 Å^–1^ indicate space group *P*4/*mbm*, Figure 3. However, the presence of the peak at 2.32 Å^–1^ indicates on the other hand space group *I*4/*mcm*. As we observe Bragg peaks expected for both the *I*4/*mcm* and *P*4/*mbm* polymorphs, this indicates long correlation lengths for the stacking of the CrF_6_ octahedra and points hence toward a two-phase coexistence of the two polymorphs. A significant improvement of the refinement is obtained when both *I*4/*mcm* and *P*4/*mbm* are used in the Rietveld refinement. Rietveld refinements and crystal structure data are given in the Supporting Information.

The relative stability of the two polymorphs was further assessed by means of DFT calculations. The energy difference between the *I*4/*mcm* and *P*4/*mbm* polymorphs is 0.19 meV/f.u. and 0.73 meV/f.u. for RbCrF_3_ and CsCrF_3_, respectively. Such narrow energy differences (Δ*E* ≪ *k*_B_ *T*) indicate that the populations of these polymorphs are comparable at room temperature. The results are thus consistent with our previous conclusions and show that the two polymorphs can coexist.

With the addition of the two new Cr­(II) fluoroperovskites, RbCrF_3_ and CsCrF_3_, the structural chemistry of *A*CrF_3_ fluoroperovskites can now be compared comprehensively, Table 1. The Goldschmidt’s tolerance factor is defined as $\tau =\frac{R_{A}+R_{X}}{\sqrt{2}\left(R_{B}+R_{X}\right)}$ , where *R*_*A*_, *R*_*B*_ and *R*_*X*_ are ionic radii of *A*, *B* and *X*, respectively, and is a measure of how various ions match in size for the perovskite arrangement, and is thereby an indicator for possible structural deformations. *A*CrF_3_ (*A* = K, NH_4_, Rb) have a tolerance factor around unity (Table 1), while NaCrF_3_ has a tolerance factor of 0.905. Values around unity would indicate high symmetry perovskite-type structures. However, the tolerance factor is not well-suited for Cr­(II) perovskites as the Jahn–Teller distortion induces an additional symmetry lowering, resulting in a tetragonal cell for *A* = K, NH_4_, Rb, Cs and a triclinic cell for NaCrF_3_. The tolerance factor of CsCrF_3_ of 1.069 is similar to that of BaTiO_3_ of 1.062, and is on the limit of the stability window of a perovskite structure. This also indicates that CsCuF_3_ will not adopt a perovskite structure (if the compound exists) due to a tolerance factor of 1.106, which is in the range where hexagonal structures are expected.

**Table 1:** Composition, Space Group, Ionic Radii of the *A*-Site Element, Unit Cell Volume per Formula Unit, Goldschmidt’s Tolerance Factor, Glazer Tilt, and Orbital Ordering Scheme for Cr­(II) Fluoroperovskites at 300 K.^a^ ^c^ ^b^

Composition	Space group	Ionic radii (Å)	Volume (Å^3^ f.u^.–1^)	τ	Glazer tilt	Orbital ordering	ref.
NaCrF_3_	*P*1̅	1.39	127.958(1)	0.905	*a* ^–^ *b* ^–^ *c* ^–^	G-type	^10^
KCrF_3_	*I*4/*mcm*	1.64	147.227(1)	0.989	*a* ^0^ *a* ^0^ *c* ^–^	G-type	^14^
NH_4_CrF_3_	*P*4_2_/*mbc*	1.67	154.677(4)	0.999	*a* ^0^ *a* ^0^ *c* ^–^	C-type	^12^
RbCrF_3_	*P*4/*mbm*	1.72	154.236(7)	1.015	*a* ^0^ *a* ^0^ *c* ^+^	C-type	This work
CsCrF_3_	*P*4/*mbm*	1.88	168.03(1)	1.069	*a* ^0^ *a* ^0^ *c* ^+^	C-type	This work
CsCrF_3_	*I*4/*mcm*	1.88	167.70(3)	1.069	*a* ^0^ *a* ^0^ *c* ^–^	G-type	This work

a The structural information is reproduced from^14^ for KCrF3, and from^12^ for NH4CrF3.

b Data for NaCrF3 is adapted with permission from^10^. Copyright 2020 American Physical Society 10.1103/PhysRevmaterials.4.054412.

c Copyright 2006 and 2025 American Chemical Society.

Interestingly, very minor differences in stability between the polymorphs manifest differently between KCrF_3_, RbCrF_3_, and CsCrF_3_. While KCrF_3_ adopts the *I*4/*mcm* structure, RbCrF_3_ adopts the *P*4/*mbm* structure with 5–10% of stacking faults of *I*4/*mcm*-type, and CsCrF_3_ is a two-phase mix between the two polymorphs. The tolerance factors of RbCrF_3_ and CsCrF_3_ are comparable to those of KCuF_3_, which contains Cu­(II) and has τ = 1.023. KCuF_3_ displays a two-phase mixture of *P*4/*mbm* and *I*4/*mcm* polymorphs, which are of similar stability.^31^ This suggests that the polymorphism originates from size effects and is not encountered for the smaller K-cation in KCrF_3_, which has a lower tolerance factor. The interplay between the *A*-cation and the Jahn–Teller effect leads to the narrow energy difference between the two polymorphs for RbCrF_3_ and CsCrF_3_.

We observe a volume expansion with increasing ionic radius of the *A*-site cation. NH_4_CrF_3_ displays a larger volume than expected due to H–F hydrogen bonding.^12^ All the compounds display distinct Jahn–Teller deformations and clear structural similarities, however, with different tilts and stacking of the CrF_6_ octahedra along the *c*-axis. NaCrF_3_ stands out owing to the small Na-cation and octahedral tilts in more directions. Also, the tetrahedral shape and the hydrogen bonding associated with the $NH_{4}^{+}$ cation have structural implications. It is worth noting that the *a* ^0^ *a* ^0^ *c* ^–^ Glazer tilt of NH_4_CrF_3_ comes from a rotation of 10^◦^, and not 90^◦^ as for the *A*CrF_3_ compounds with the same tilt system, Table 1.

Finally, we can compare the Cr­(II) fluoroperovskites in terms of the Van Vleck distortion modes.^32^ These modes describe the octahedral displacements and are particularly useful for Jahn–Teller distorted octahedra. The *Q*_2_ and *Q*_3_ modes describe a planar rhombic distortion (orthorhombic distortion where the two opposite bonds in the *xy*-plane lengthen, and the other two shorten) and a tetragonal distortion (elongation or compression of the axial bonds), respectively. The modes are defined as

1 $$Q_{2}=l-s$$

2 $$Q_{3}=\frac{\left(2m-l-s\right)}{\sqrt{3}}$$

with *s*, *m*, and *l* being the short, medium, and long bond distances, respectively. The magnitude of the distortion is defined as

3 $$\rho_{0}={\left(Q_{2}^{2}+Q_{3}^{2}\right)}^{1/2}$$

while the angle is

4 $$\phi =\arctan\left(\frac{Q_{2}}{Q_{3}}\right)$$

The angle is not a physical angle, but rather the angle between the different orbital states. The magnitude and angle of the distortions were calculated for all *A*CrF_3_, using the VanVleckCalculator.^33^ The results are presented in the polar plot in Figure 5. In this plot, the occupation of the 3*z* ^2^-*r* ^2^, 3*y* ^2^-*r* ^2^, and 3*x* ^2^-*r* ^2^ orbitals for 2-fold degenerate *e* orbitals correspond to 0^◦^, 120^◦^, and 240^◦^, respectively. For *A*CrF_3_, all the compounds lie close to 120^◦^. This result therefore clearly shows that the occupied orbital on Cr­(II) is 3*y* ^2^-*r* ^2^. This corresponds to a dominant *Q*_3_ Van Vleck mode with elongation of the octahedra and a minor planar rhombic distortion. The mean of the angle for the six compounds is 122^◦^. For CsCrF_3_, the change from *I*4/*mcm* to *P*4/*mbm* reduces the magnitude of the distortion slightly, and shifts the angle from slightly below 120^◦^ to slightly above. No particular trend is observed between the magnitude of the distortion and the size of the *A*-site cation.

**Figure 5:**
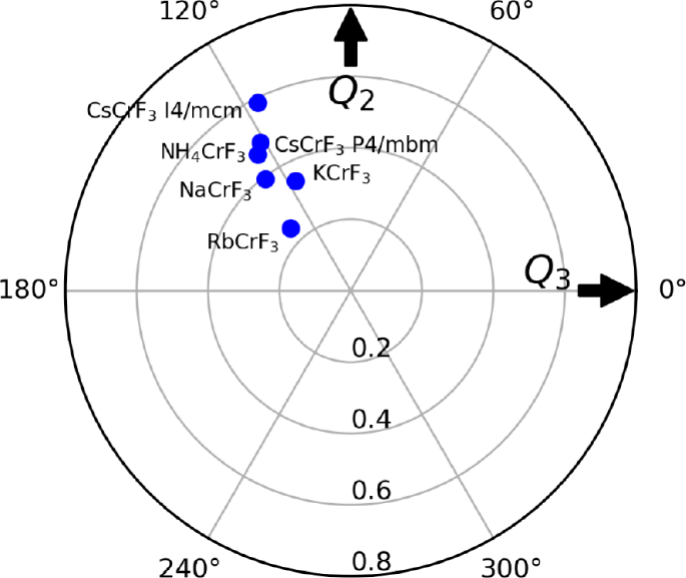
Radial plot of the bond distortion modes *Q*_2_ and *Q*_3_ for ACrF_3_. The structural information is reproduced from ref.^14^ for KCrF_3_, and from ref^12^ for NH_4_CrF_3_. Copyright 2006 and 2025 American Chemical Society. Data for NaCrF_3_ is adapted with permission from ref^10^ copyright 2020 American Physical Society 10.1103/PhysRevMaterials.4.054412.

### Magnetic Properties

At high temperatures, the DC magnetic susceptibility χ­(*T*) of both RbCrF_3_ and CsCrF_3_ behave as typical Curie–Weiss (CW) paramagnets, Figure 6. From CW fits in the range 150–300 K, we extract an effective paramagnetic moment of 4.65 μ_B_ and 4.68 μ_B_ for RbCrF_3_ and CsCrF_3_, respectively. The values are in good agreement with the theoretical μ_eff_ = 4.9 μ_B_ for a high-spin Cr^2+^ (*d* ^4^) *S* = 2 system. The negative Weiss temperatures of −12 K for both compounds indicate that antiferromagnetic correlations dominate in the paramagnetic regime.

**Figure 6:**
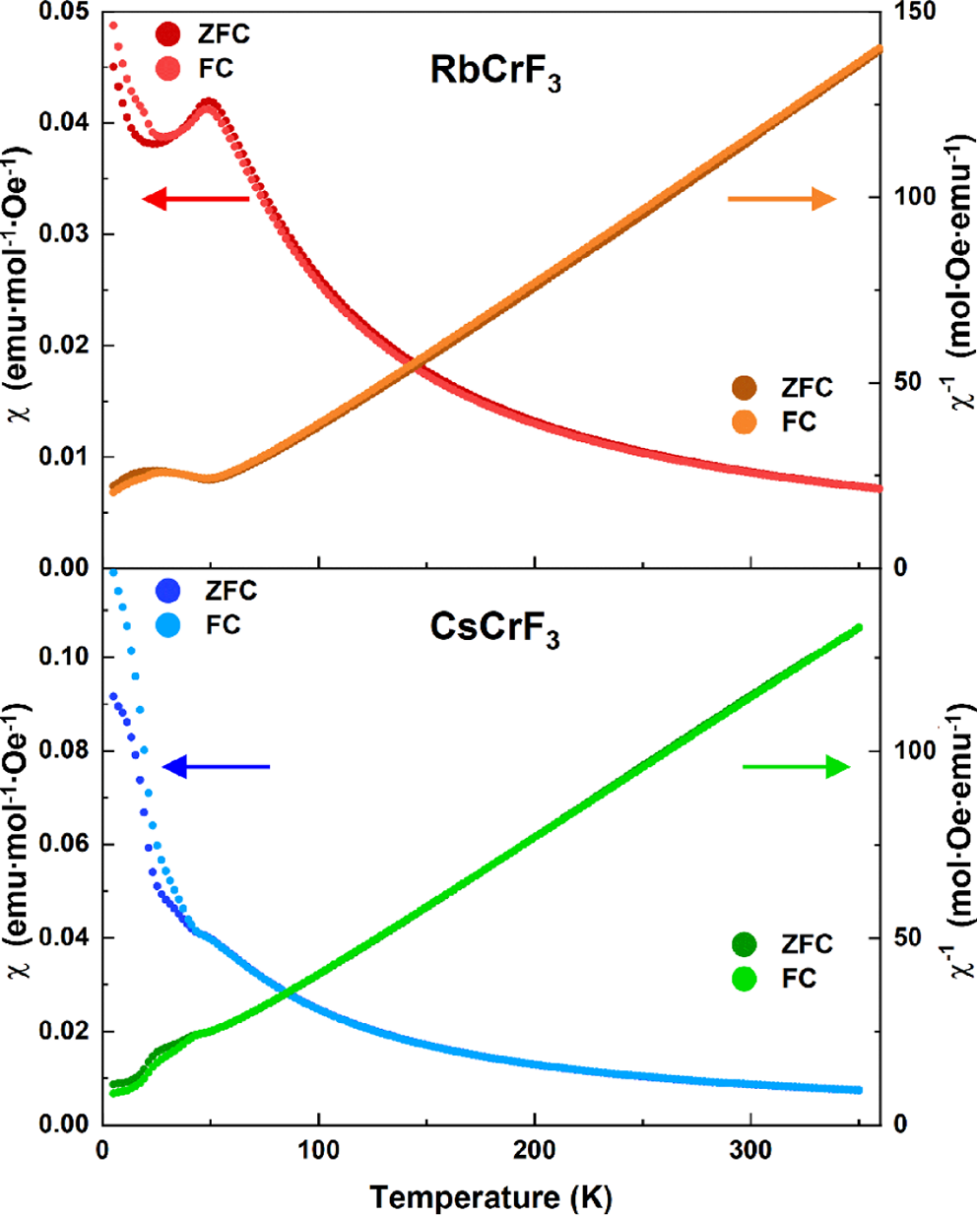
χ­(*T*) Zero field cooled (ZFC) and field cooled (FC) data for RbCrF_3_ (top) and CsCrF_3_ (bottom); measured in a field of 500 Oe.

At low temperatures, RbCrF_3_ and CsCrF_3_ show a distinct transition to a long-range antiferromagnetic state with Néel temperatures (*T*_*N*_) of 50 and 47 K, respectively. For both compounds, a difference is seen between the ZFC and FC data samples below *T*_*N*_, which indicates a possible spin canting induced by the applied field. However, we cannot exclude the possibility of a glassy magnetic component such as spin-glass-like behavior or magnetic clustering. For CsCrF_3_, a small second anomaly is observed at 20 K. A similar anomaly is observed for KCrF_3_ in refs. 14,34 which was suggested to originate from weak ferromagnetism or an incommensurate-commensurate transition. However, the moderate change in susceptibility points rather to a low temperature weakly canted AFM state. Further experiments beyond the scope of this work are needed to address this. Isothermal *M*(*H*) measurements at 5 K agree with an antiferromagnetic behavior for both samples, see Supporting Information. A negligible opening is observed at low positive values of the magnetic field in line with the difference between the FC and ZFC data.

The magnetic properties of RbCrF_3_ and CsCrF_3_ align well with those of other *A*CrF_3_ fluoroperovskites. The Néel temperatures are between 45 and 60 K for the tetragonal compounds, while it is 21.3 K for triclinic NaCrF_3_. The lower Néel temperature of NaCrF_3_ is justified based on the distorted crystal structure, since the superexchange interactions weaken when the Cr–F–Cr bond angles deviate from 180^◦^. Based on the orbital ordering scheme resulting from the Jahn–Teller effect, we expect an A-type antiferromagnetic structure. This is experimentally shown for NH_4_CrF_3_, NaCrF_3_, and KCrF_3_ by neutron powder diffraction and would comply with the Goodenough-Kanamori-Anderson rules.^10,12,34,35^

## Conclusion

Here, we report for the first time the crystal structures and magnetic properties of RbCrF_3_ and CsCrF_3_. The compounds were prepared using a wet chemical technique, which has proved reliable for preparing Cr­(II) fluoroperovskites. RbCrF_3_ is best described by the *P*4/*mbm* space group, but the weak intensity of the (211) peak suggests 5–10% stacking faults in the rotation of the distorted CrF_6_ along the *c*-axis (*I*4/*mcm*-type stacking). CsCrF_3_ displays a two-phase coexistence of *I*4/*mcm* and *P*4/*mbm* phases, similar to what reported for KCuF_3_. The structural properties of these new Cr­(II) fluoroperovskites result from the interplay between the size of the *A*-site cation and the Jahn–Teller effect induced by the *d* ^4^ electron configuration of Cr­(II). RbCrF_3_ and CsCrF_3_ have Néel temperatures of 50 and 47 K, and effective paramagnetic moments of 4.65 μ_B_ and 4.68 μ_B_, respectively. These values are consistent with a high-spin *S = 2* system and with other Cr­(II) fluoroperovskites.

## Supplementary Material



## Notes

Structural, optical, and magnetic data for Cr­(II) fluoroperovskites, with figures and tables detailing Rietveld refinements, and magnetization behavior (PDF)
