# Supplementary material for: ACrF3 Jahn–Teller-Distorted Fluoroperovskites: Expanding to RbCrF3 and CsCrF3
Source: Inorg Chem. 2025 Sep 4;64(36):18316–22. doi: 10.1021/acs.inorgchem.5c02668 (PMC12442058; doi:10.1021/acs.inorgchem.5c02668)
Supplement: Supplementary file 1 [file ic5c02668_si_001.pdf]

# $A$CrF$_3$ Jahn-Teller Distorted Fluoroperovskites: Expanding to $RbCrF_3$ and $CsCrF_3$

Øystein S. Fjellvåg,[1,*] Heesoo Park,[2] Fabien Veillon,[3] Jike Lyu,[4,5] Marisa Medarde,[4] Salah B. Amedi,[2] Fabian L. M. Bernal,[6] Helmer Fjellvåg,[2] Bjørn C. Hauback,[1] and Bruno Gonano[2]

[1]*Department for Hydrogen Technology, Institute for Energy Technology, PO Box 40, NO-2027, Kjeller, Norway*
[2]*Chemistry Department and Center for Material Science and Nanotechnology, University of Oslo, NO-0315, Norway*
[3]*Laboratory Crismat, UMR6508 CNRS, Normandie University, ENSICAEN, UNICAEN, 6 bd Maréchal Juin, 1450 Caen cedex 4, France*
[4]*Laboratory for Multiscale Materials Experiments, Paul Scherrer Institut, CH-5232 Villigen-PSI, Switzerland*
[5]*CAS Key Laboratory of Magnetic Materials and Devices, Ningbo Institute of Materials Technology and Engineering, Chinese Academy of Sciences, Ningbo 315201, China*
[6]*Division for Research, Dissemination and Education, IT-department, University of Oslo, Oslo, Norway*

## I. SUPPORTING INFORMATION

The Supporting Information contains:

- Figure S1 show the optical data for all Cr(II) fluoroperovskites.
- Table S1 contains the energy of the $E_4$ and $E_5$ transitions.
- Figure S2, Figure S3, and Figure S4 show the Rietveld refinements of $RbCrF_3$ in space group $P4/mbm$ and $CsCrF_3$ in space groups $P4/mbm$ and $I4/mcm$.
- Figure S5 show Rietveld refinement of $CsCrF_3$ with both space groups $P4/mbm$ and $I4/mcm$.
- Table S2, Table S3', Table S4 and Table S5 contains structural information for $RbCrF_3$ and $CsCrF_3$.
- Table S6 and Table S7 compare structural features for Cr(II) fluoroperovskites.
- Figure S6 shows the ionic radii vs volume.
- Figure S7 shows the magnetization curves for $RbCrF_3$ and $CsCrF_3$ at 5 K.
- Table S8 compares the magnetic properties of Cr(II) fluoroperovskites.

[1] F.L.M. Bernal, J. Sottmann, D.S. Wragg, H. Fjellvåg, Ø.S. Fjellvåg, C. Drathen, W.A. Sławiński, and O.M. Løvvik Structural and magnetic characterization of the elusive Jahn-Teller active $NaCrF_3$ *Phys. Rev. Materials*, **2020**, 4, 054412.
[2] S. Margadonna and G. Karotsis Cooperative Jahn-Teller Distortion, Phase Transitions, and Weak Ferromagnetism in the $KCrF_3$ Perovskite *J. Am. Chem. Soc.*, **2006**, 128, 16436–16437.
[3] Ø. S. Fjellvåg, B. Gonano, F. L. M. Bernal, S. B. Amedi, J. Lyu, V. Pomjakushin, M. Medarde, D. Chernyshov, K. Marshall, M. Valldor, H. Fjellvåg, and B. C. Hauback Order-to-Disorder Transition and Hydrogen Bonding in the Jahn–Teller Active $NH_4CrF_3$ Fluoroperovskite *Inorg. Chem.*, **2024**, 63, 10594-10602.

* Corresponding author: oystein.fjellvag@ife.no

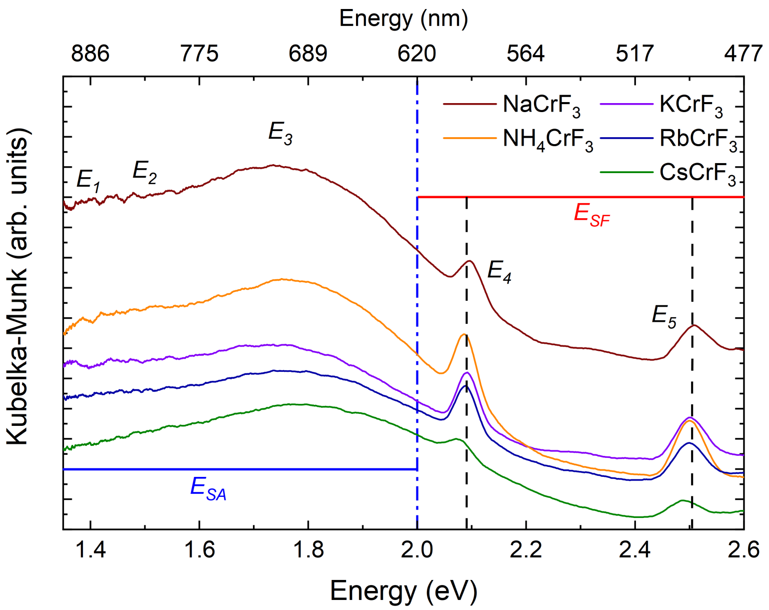


FIG. S1: Optical absorption spectra of $A\mathrm{CrF_3}$ ($A$ = Na, K, Rb, Cs, $NH_4$) collected at room temperature. The spin-allowed (SA) transitions are labeled $E_1$-$E_3$, while $E_4$ and $E_5$ are spin-forbidden (SF).

TABLE S1: Spin-forbidden transition transitions for $A\mathrm{CrF_3}$.

| Composition | $E_4$ (eV) | $E_5$ (eV) |
|---|---|---|
| $NaCrF_3$ | 2.1024(9) | 2.5016(15) |
| $KCrF_3$ | 2.0932(3) | 2.5001(5) |
| $NH_4CrF_3$ | 2.0896(3) | 2.4972(5) |
| $RbCrF_3$ | 2.0903(3) | 2.4974(5) |
| $CsCrF_3$ | 2.0788(8) | 2.4890(9) |

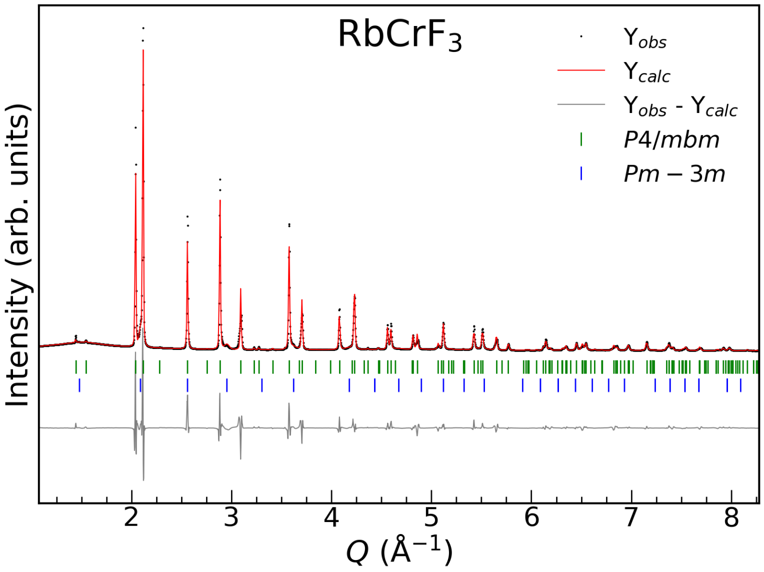


FIG. S2: Measured, calculated, and difference intensity curves for Rietveld refinement of tetragonal $RbCrF_3$ ($P4/mbm$) as the main phase, with a small (7.8(8) w%) secondary cubic phase ($Pm\bar{3}m$) with $\lambda$=0.25509 Å at room temperature. $R_{wp}$ for the refinement is 14.339 %.

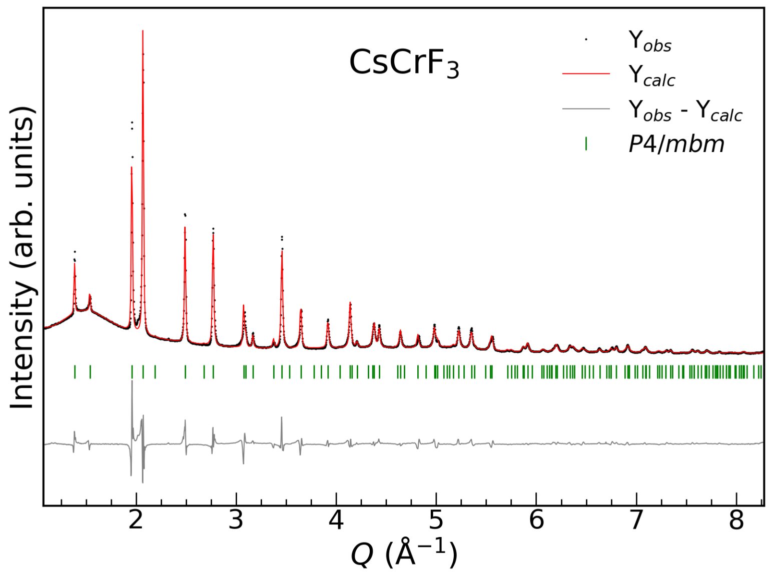


FIG. S3: Measured, calculated, and difference intensity curves for Rietveld refinement of tetragonal $CsCrF_3$ ($P4/mbm$) with $\lambda$=0.25509 Å at room temperature. The low-angle hump for the background is from the capillary. $R_{wp}$ for the refinement is 6.075 %.

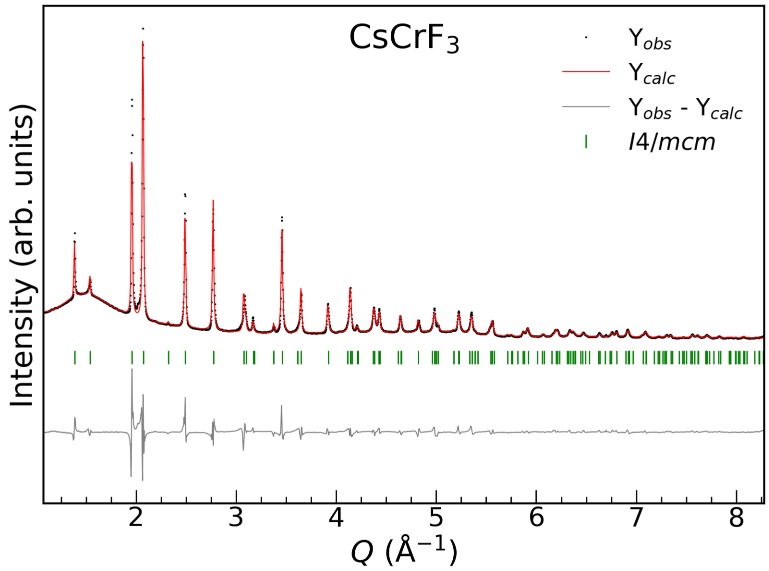


FIG. S4: Measured, calculated, and difference intensity curves for Rietveld refinement of tetragonal $CsCrF_3$ ($I4/mcm$) with $\lambda$=0.25509 Å at room temperature. The low-angle hump for the background is from the capillary. $R_{wp}$ for the refinement is 6.067 %.

TABLE S2: Atomic coordinates of $RbCrF_3$ from Rietveld refinement of X-ray ($\lambda$=0.25448 Å) data at room temperature in space group $P4/mbm$ with $a = b = 6.16111(11)$ Å, and $c = 4.06318(10)$ Å. Occupancy values have been fixed to 1.

| Atom | Wyckoff site | $x$ | $y$ | $z$ | Occ | B $_{iso}$ (Å$^2$) |
|---|---|---|---|---|---|---|
| Rb1 | 2b | 0 | 0 | 1/2 | 1 | 1.03(5) |
| Cr1 | 2d | 0 | 1/2 | 0 | 1 | 1.26(8) |
| F1 | 2c | 0 | 1/2 | 1/2 | 1 | 1.12(18) |
| F2 | 4g | 0.240(3) | 0.740(3) | 0 | 1 | 2.04(19) |

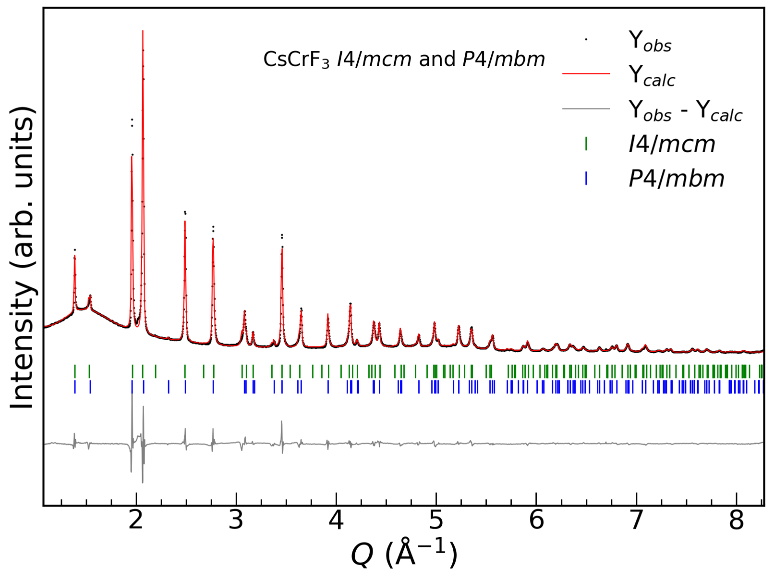


FIG. S5: Measured, calculated, and difference intensity curves for Rietveld refinement of both $I4/mcm$ and $P4/mbm$ $CsCrF_3$ with $\lambda$=0.25509 Å at room temperature. The low-angle hump for the background is from the capillary. $R_{wp}$ for the refinement is 4.094 %.

TABLE S3: Atomic coordinates of the secondary cubic $RbCrF_3$ phase from Rietveld refinement of X-ray ($\lambda$=0.25448 Å) data at room temperature in space group $Pm\bar{3}m$ with $a = b = c = 4.2509(12)$ Å. Occupancy values have been fixed to 1.

| Atom | Wyckoff site | $x$ | $y$ | $z$ | Occ | B $_{iso}$ (Å$^2$) |
|---|---|---|---|---|---|---|
| Rb1 | 1b | 0.5 | 0.5 | 0.5 | 1 | 1 |
| Cr1 | 1a | 0 | 0 | 0 | 1 | 1 |
| F1 | 3d | 0 | 0.5 | 0 | 1 | 1 |

TABLE S4: Atomic coordinates of $CsCrF_3$ from Rietveld refinement of X-ray ($\lambda$=0.25448 Å) data at room temperature in space group $P4/mbm$ with $a = b = 6.4136(2)$ Å, and $c = 4.0848(2)$ Å. Occupancy values have been fixed to 1.

| Atom | Wyckoff site | $x$ | $y$ | $z$ | Occ | B$_{iso}$ (Å$^2$) |
|---|---|---|---|---|---|---|
| Rb1 | 2b | 0 | 0 | 1/2 | 1 | 1.05(6) |
| Cr1 | 2d | 0 | 1/2 | 0 | 1 | 2.2(1) |
| F1 | 2c | 0 | 1/2 | 1/2 | 1 | 1.9(3) |
| F2 | 4g | 0.229(3) | 0.729(3) | 0 | 1 | 3.0(4) |

TABLE S5: Atomic coordinates of $CsCrF_3$ from Rietveld refinement of X-ray ($\lambda$=0.25448 Å) data at room temperature in space group $I4/mcm$ with $a = b = 6.4103(4)$ Å, and $c = 8.1624(9)$ Å. Occupancy values have been fixed to 1.

| Atom | Wyckoff site | $x$ | $y$ | $z$ | Occ | B$_{iso}$ (Å$^2$) |
|---|---|---|---|---|---|---|
| Cs1 | 4a | 0 | 0 | 0.5 | 1 | 1.0(1) |
| Cr1 | 4d | 0 | 0.5 | 0 | 1 | 1.8(3) |
| F1 | 4b | 0 | 0.5 | 0.25 | 1 | 1.8(7) |
| F2 | 8h | 0.279(4) | 0.779(4) | 0 | 1 | 1.7(7) |

TABLE S6: Comparison of lattice parameters for Cr(II) fluoroperovskites. * $NaCrF_3$ is triclinic with $\alpha = 90.5039(3)^\circ$, $\beta = 92.2554(3)^\circ$, and $\gamma = 86.0599(2)^\circ$[1]. The structural information is reproduced from ref. [2] for $KCrF_3$, and from ref [3] for $NH_4CrF_3$. Copyright 2006 and 2025 American Chemical Society. Data for $NaCrF_3$ is adapted with permission from ref [1] copyright 2020 American Physical Society https://doi.org/10.1103/PhysRevMaterials.4.054412.

| Composition | Space group | $a$ (Å) | $b$ (Å) | $c$ (Å) | Volume (Å$^3$) | Ref. |
|---|---|---|---|---|---|---|
| $NaCrF_3$* | $P\bar{1}$ | 5.51515(2) | 5.68817(3) | 8.18349(3) | 255.915(2) | [1] |
| $KCrF_3$ | $I4/mcm$ | 6.05230(2) | 6.05230(2) | 8.02198(4) | 294.453(2) | [2] |
| $NH_4CrF_3$ | $P4_2/mbc$ | 6.22593(6) | 6.22593(6) | 7.96165(8) | 299.522(4) | [3] |
| $RbCrF_3$ | $P4/mbm$ | 6.16111(11) | 6.16111(11) | 4.06318(10) | 154.236(7) | This work |
| $CsCrF_3$ | $P4/mbm$ | 6.4136(2) | 6.4136(2) | 4.0848(2) | 168.028(14) | This work |
| $CsCrF_3$ | $I4/mcm$ | 6.4103(4) | 6.4103(4) | 8.1624(9) | 335.40(6) | This work |

TABLE S7: Distortion index ($D = \frac{1}{n}\sum_{i=1}^{n}\frac{|l_i - l_{av}|}{l_{av}}$, where $l_i$ is the distance from the central atom to the $i$th coordinating atom, and $l_{av}$ is the average bond length) and quadratic elongation ($\lambda = \frac{1}{n}\sum_{i=1}^{n}(\frac{l_i}{l_0})^2$, where $l_0$ is the center-to-vertex distance) for Cr(II) fluoroperovskites at 300 K. For $NaCrF_3$, we have listed all Cr sites. The structural information is reproduced from ref. [2] for $KCrF_3$, and from ref [3] for $NH_4CrF_3$. Copyright 2006 and 2025 American Chemical Society. Data for $NaCrF_3$ is adapted with permission from ref [1] copyright 2020 American Physical Society https://doi.org/10.1103/PhysRevMaterials.4.054412.

| Composition | Site | Distortion index | Quadratic elongation | $s$ Cr-F | $m$ Cr-F | $l$ Cr-F | Ref. |
|---|---|---|---|---|---|---|---|
| $NaCrF_3$ | Cr1 | 0.07837 | 1.0209 | 1.987(5) | 2.028(5) | 2.383(6) | [1] |
| $NaCrF_3$ | Cr2 | 0.05901 | 1.0094 | 1.976(5) | 2.045(5) | 2.289(5) | [1] |
| $NaCrF_3$ | Cr3 | 0.07235 | 1.0173 | 1.986(5) | 2.019(5) | 2.346(5) | [1] |
| $NaCrF_3$ | Cr4 | 0.07686 | 1.0181 | 1.986(5) | 2.022(5) | 2.371(5) | [1] |
| $KCrF_3$ | Cr1 | 0.06327 | 1.0089 | 1.986(4) | 2.00549(1) | 2.294(4) | [2] |
| $NH_4CrF_3$ | Cr1 | 0.08611 | 1.0166 | 1.9913(5) | 2.003(2) | 2.418(2) | [3] |
| $RbCrF_3$ | Cr1 | 0.04259 | 1.0043 | 2.03159(6) | 2.09(3) | 2.27(3) | This work |
| $CsCrF_3$ $P4/mbm$ | Cr1 | 0.08074 | 1.0146 | 2.04240(11) | 2.08(3) | 2.46(3) | This work |
| $CsCrF_3$ $I4/mcm$ | Cr1 | 0.10289 | 1.0236 | 2.00(4) | 2.0406(3) | 2.53(4) | This work |

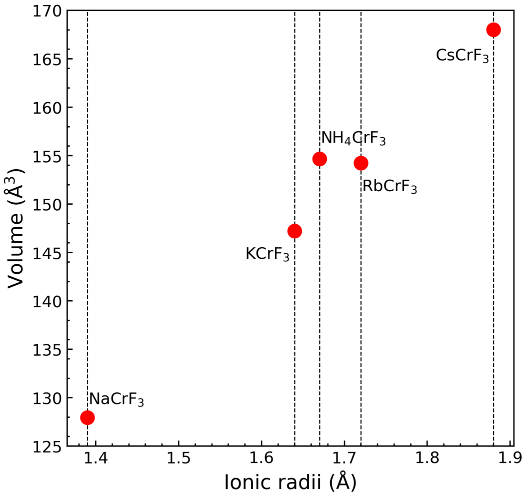


FIG. S6: Volume per formula unit of Cr(II) fluoroperovskites as a function of the $A$-site ionic radii.

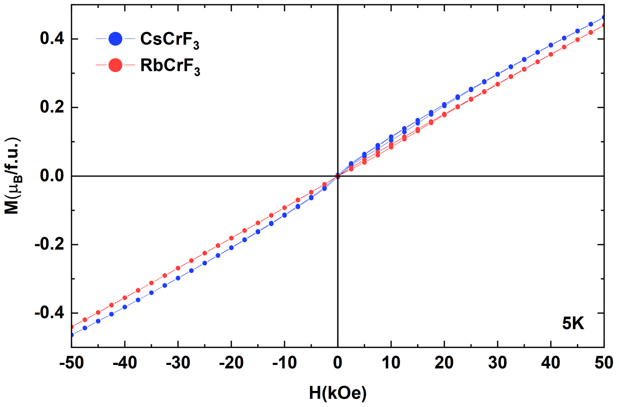


FIG. S7: $M(H)$ curves for $RbCrF_3$ and $CsCrF_3$ collected at 5 K.

TABLE S8: Comparison of magnetic properties of $A$CrF$_3$. The magnetic information is reproduced from ref. [2] for $KCrF_3$, and from ref [3] for $NH_4CrF_3$. Copyright 2006 and 2025 American Chemical Society. Data for $NaCrF_3$ is adapted with permission from ref [1] copyright 2020 American Physical Society https://doi.org/10.1103/PhysRevMaterials.4.054412.

| Composition | $T_N$ (K) | $\mu_{eff}$ ($\mu_B$) | $\theta$ (K) | Ref. |
|---|---|---|---|---|
| $RbCrF_3$ | 50 | 4.65 | -12 | This work |
| $CsCrF_3$ | 47 | 4.68 | -12 | This work |
| $NH_4CrF_3$ | 60 | 4.3 | -33 | [3] |
| $KCrF_3$ | 46 | 4.7 | 2.7 | [2] |
| $NaCrF_3$ | 21.3 | 4.47 | -4 | [1] |
